# Brinell-Hardness data (HBW 2.5/62.5) of aluminum alloy EN AW-2618A after different aging times and temperatures

**DOI:** 10.1016/j.dib.2022.108830

**Published:** 2022-12-19

**Authors:** Christian Rockenhäuser, Philipp von Hartrott, Birgit Skrotzki

**Affiliations:** a Bundesanstalt für Materialforschung und -prüfung (BAM), Unter den Eichen 87, 12205 Berlin, Germany; b Fraunhofer-Institut für Werkstoffmechanik (IWM), Wöhlerstraße 11, 79108 Freiburg, Germany

**Keywords:** Aluminum alloy, EN AW-2618A, Brinell hardness, Aging, Creep, Ostwald ripening, Reheating

## Abstract

The article covers data on the Brinell hardness of the forged precipitation-hardened aluminum alloy EN AW-2618A in the initial T61 condition (i. e. slightly underaged) and after isothermal aging for up to 25,000 h at aging temperatures between 160 °C and 350 °C. In addition, the hardness was determined on specimens after creep testing at 190 °C and various stresses. The hardness decreases with increasing aging time due to the microstructural evolution of the hardening precipitates. The drop occurs faster the higher the aging temperature. Aging under creep load additionally accelerates the hardness decrease.

**Specifications Table**

**Table:** 

Subject	Materials Science; Engineering
Specific subject area	Material Characterization; Metals and Alloys;
Type of data	Table (Microsoft Excel file format) Graph
How the data were acquired	Hardness testers: Emco Test M4C 025 G3 and Wolpert DIATestor 2RC
Data format	Raw Analyzed
Description of data collection	Brinell hardness (HBW 2.5/62.5) was measured according to DIN EN 6506-1 on aged platelets and on longitudinal sections of creep test pieces after creep testing, respectively. The surfaces were ground and polished before testing.
Data source location	Bundesanstalt für Materialforschung und -prüfung (BAM) Berlin Germany Fraunhofer-Institut für Werkstoffmechanik (IWM) Freiburg Germany
Data accessibility	Repository name: Zenodo Data identification number: 10.5281/zenodo.6787084
Related research article	C. Rockenhäuser, S. Schriever, P. von Hartrott, B. Piesker, B. Skrotzki: Comparison of Long-Term Radii Evolution of the S-phase in Aluminum Alloy 2618A During Ageing and Creep. Mater Sci Eng A 716 (2018) 78-86; 10.1016/j.msea.2018.01.033 C. Rockenhäuser, C. Rowolt, B. Milkereit, R. Darvishi Kamachali, O. Kessler, B. Skrotzki, On the Long-Term Aging of S-Phase in Aluminum Alloy 2618A, J Mater Sci 56 (2021) 8704–8716; 10.1007/s10853-020-05740-x

## Value of the Data

• The data include very long aging times of up to 25,000 h, which represent realistic operating times of heavy engine components.
• The data can be used to build time- and temperature-dependent models of mechanical strength of aluminum, which are necessary for numerical component assessment.
• The dataset can be used to calibrate and validate numerical thermodynamic and thermokinetic models.
• Hardness data can be correlated with microstructural data given in [1], [2], [3].
• The data can be used for comparative material selection processes.

## 1 Data Description

The data presented here was the basis of two publications [1,2] and also includes data on additional aging temperatures and times.

• The spreadsheet “Average” of the Excel file “Brinell_hardness_ENAW2618A.xlsx” in the repository [4] summarizes the average values of the 5 individual measurements of Brinell hardness HBW 2.5/62.5 of all investigated aging and creep conditions, respectively.
• The values of the 5 individual hardness measurements (*d*_1_, *d*_2_) for the load-free aging at 160 °C, 180 °C, and 190 °C are provided in the spreadsheet “Individual values” in [4]. For aging times see section “Experimental design, materials, and methods” in this manuscript.
• The spreadsheet “Individual values creep” summarizes the data of the specimens which were aged under applied load in [4]. The parameters of the creep tests are detailed in Table 2.

The results of the chemical analysis of the studied alloy are given in Table 1.

**Table 1:** Actual chemical composition of alloy EN AW-2618A [2].

Element	Cu	Mg	Fe	Ni	Si	Mn	Zn	Ti	Al
wt.%	2.5	1.6	1.1	1.1	0.24	< 0.1	< 0.1	0.06	Balance

**Fig. 1:**
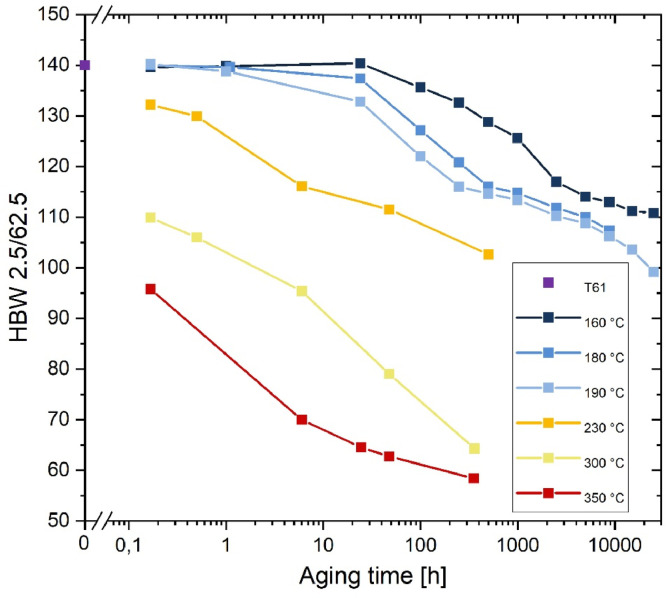
Brinell hardness as a function of aging time and temperature (load free aging). T61 represents a slightly underaged initial condition.

Fig. 1 shows the development of hardness value for load-free aging as a function of aging temperature and (logarithm of) aging time. The colors correspond to the different temperatures. As expected, the hardness decreases with increasing aging time for all temperatures due to microstructural degradation of the optimized precipitate microstructure in the T61 state. The hardness curve for *T* = 190 °C (light blue) shows an initially fast decrease from 140 HBW (T61 condition) to 116 HBW after 250 h. The hardness decrease then slows down with increasing time, and a final hardness of 99.2 HBW is measured after 25,000 h of aging. The hardness loss is due to the microstructural evolution of the nm-sized S-phase Al_2_CuMg, which is the strengthening phase in this Al-alloy. We have shown in [1,2] for this aging temperature, that the average radii of the S-phase increase with aging time and the hardening phase coarsens. Initially, the increase is very rapid, followed by a pronounced change in slope after 1,000 hours of aging, which is due to the initial growth kinetics of the precipitates. The coarsening kinetics slow down considerably thereafter.

Higher temperatures result in a faster decrease of the hardness. This is particularly pronounced for all temperatures exceeding the age-hardening temperature of the alloy (i. e. 230 °C, 300 °C and 350 °C, orange, yellow and red curves in Fig. 1). Here, a significant to dramatic drop in hardness can be observed after only 10 min (from 140 HBW in T61 state to 132 HBW, 110 HBW and 95.8 HBW, respectively). Although there are no corresponding microstructure studies for these temperatures, it can be reasonably assumed that the coarsening processes of the S-phase are strongly accelerated above 200 °C due to faster diffusion and consequently the hardening effect is rapidly lost.

In contrast to this, it is noticeable that the aging temperature 160 °C (dark blue curve), which is 35 K below the initial age-hardening temperature, results in a significantly lower drop in hardness, indicating that the microstructural degradation processes are much slower than at 180 °C and 190 °C: The coarsening of the S-phase proceeds more slowly with ageing at 160 °C than at 190 °C [5] due to slower diffusion process.

**Fig. 2:**
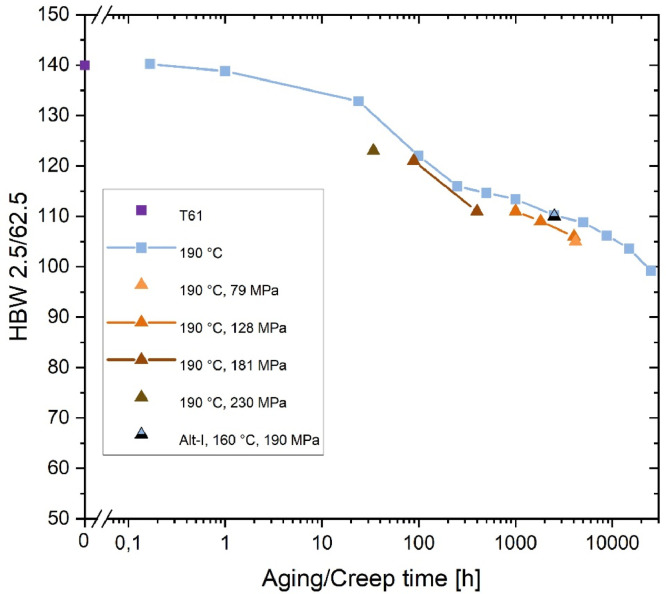
Brinell hardness as a function of aging time or creep time at *T* = 190 °C. T61 represents a slightly underaged initial condition. The creep specimen "Alt I" was pre-aged at 190 °C for 1,000 h and subsequently deformed in the creep test at 160 °C and 190 MPa.

Fig. 2 shows the development of hardness value for *T* = 190 °C with (logarithm of) time. The light blue squares represent data for load free aging (same as in Fig. 1), the triangles for aging with an externally applied load (i. e. creep conditions). The different colors of the triangles represent different applied loads. In the case of the creep tests, the hardness decrease is several HBW larger than for the same aging time without external load. The corresponding microstructure investigations showed that the mean radii of the S phase of the creep specimens grow significantly faster than the load-free specimens aged at the same temperature, suggesting an accelerated coarsening under load [2,5]. The creep specimen "Alt I" (black triangle) was pre-aged at 190 °C for 1,000 h and subsequently deformed in the creep test at 160 °C and 190 MPa. The data point fits significantly better with the results of the specimens aged stress-free at 190 °C than at 160 °C (cf. Fig. 1). This indicates that pre-aging at the higher temperature has a significantly stronger influence on the degradation processes than subsequent creep deformation at the lower temperature. (Note: for the Alt I specimen, the time of the pre-aging and the creep test were summed up).

## 2 Experimental Design, Materials and Methods

### 2.1 Material

The material was received as forged circular compressor wheel blanks (diameter ca. 195 mm, height ca. 130 mm) in T61 condition according to DIN EN 515 [6] with a chemical composition corresponding to DIN EN 573 [7] as shown in Table 1.

**Fig. 3:**
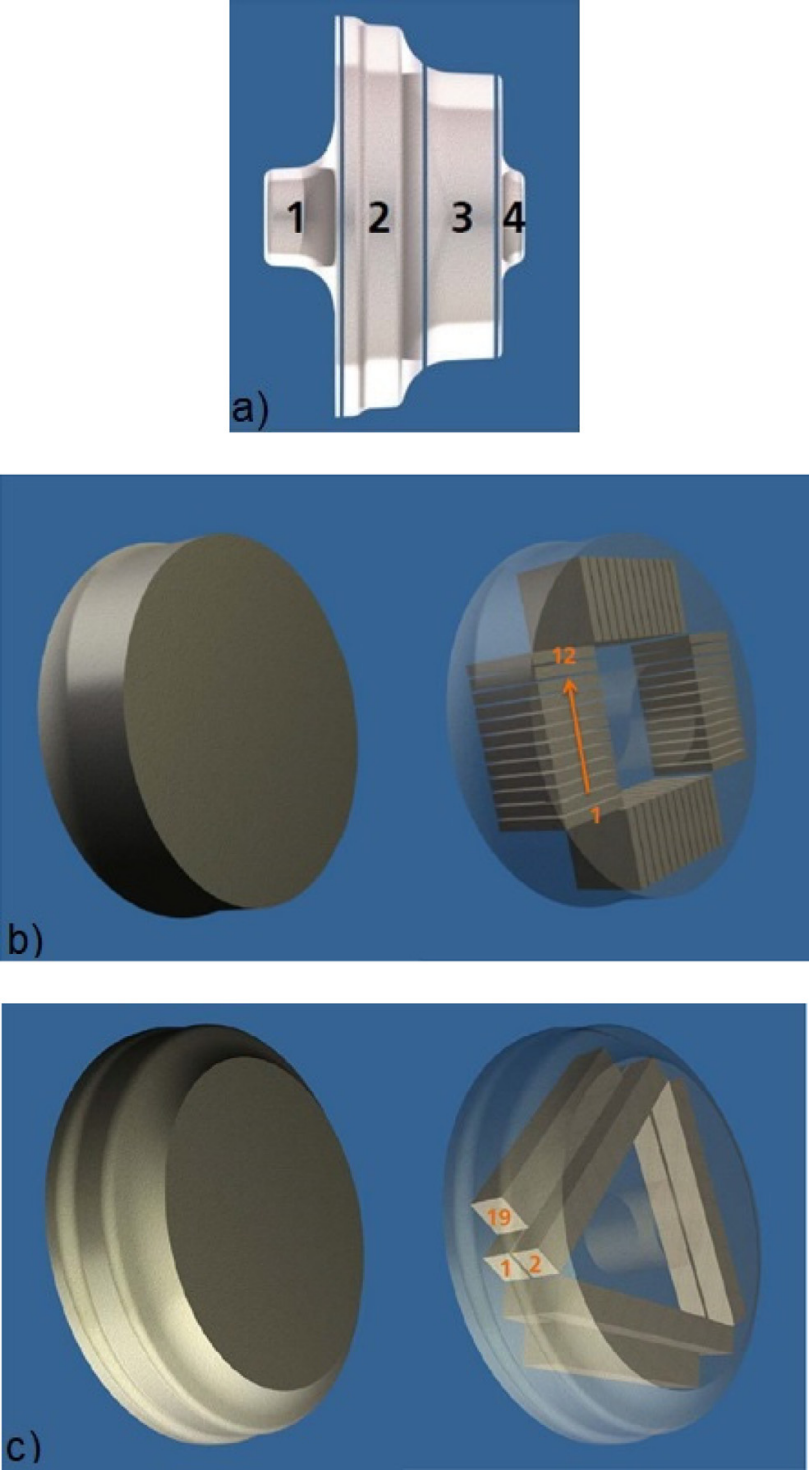
a) Forged disk with segments. Sampling plan of b) the hardness testing platelets from the circular disc Section 3, and c) the creep test pieces from Section 2. [5].

The forged disc consists of 4 segments, Fig. 3a). The hardness platelets (30 mm × 30 mm x 4 mm) were taken from segment 3. Sampling is schematically shown in Fig. 3b). The T61 state represents a slightly underaged condition. The T61 heat treatment includes a solution heat treatment at 530 °C for 8 h, followed by quenching into boiling water and ageing at 195 °C for 28 h.

### 2.2 Aging treatments

Isothermal aging treatments were performed on the hardness platelets (starting in the T61 state) for different times (10 min to 25,000 h) and temperatures (160 °C, 180 °C, 190 °C). The temperature constancy of the long-term ageing was on average significantly better than ± 2 °C, with a total of less than 10 short-term overshoots of up to 4 °C. In addition, ageing treatments at higher temperatures (230 °C, 300 °C, 350 °C) were carried out for up to 500 h.

Creep tests were conducted on cylindrical creep test pieces with a gauge length diameter of 6 mm. The test pieces were extracted from the blank (Section 2 in Fig. 3a), orthogonal to the forging direction, specimen position 19 in Fig. 3c)). These samples were subjected to creep testing with parameters as given in Table 2. A few tests were run to fracture of the test piece and time to fracture is given. Most of the tests were interrupted (time to interruption given). The test pieces were in the T61 state before creep testing, except for the last condition, where the test piece was overaged for 1,000 h at 190 °C to simulate long term operation condition.

**Table 2:** Creep test parameters and material state before testing.

Condition	T [ °C]	σ [MPa]	t_fracture_ [h]	t_interrupt_ [h]
T61	190	230	33.9	-
T61	190	181	-	88.6
T61	190	181	400	-
T61	190	128	-	1002
T61	190	128	-	1820
T61	190	128	-	4026
T61	190	79	-	4172
T61 + 1000 h/ 190 °C	160	190	1515	-

### 2.3 Brinell hardness measurements

The hardness measurements were taken on square bulk samples (30 mm × 30 mm, thickness 4 mm). The specimens with ageing temperatures of 160 °C, 180 °C and 190 °C were tested at BAM according to DIN EN 6506-1 [8] using an Emco Test M4C 025 G3 hardness tester. The test load was *F* = 612.916 N, the ball diameter *D* = 2.5 mm and the penetration time 10 s - 15 s. On each specimen, the hardness was determined as the average of 5 individual measurements (distances between sample edge and indentations was ≥ 3 mm, and between indentations ≥ 3.5 mm).

To measure the hardness of the creep sample after creep loading, the samples were cut in longitudinal direction and the first undeformed position nearest to the center of the creep samples was used for the measurement.

The surfaces were previously ground and polished.

The specimens with aging temperatures > 190 °C were tested at IWM. A Wolpert DIATestor 2RC hardness tester was used. The test load was *F* = 612.916 N, the ball diameter *D* = 2.5 mm and the penetration time 4 s - 6 s. The hardness values were determined in the same way as at BAM.

According to DIN EN 6506-1 [8] the Brinell hardness HBW is calculated as:

$$HBW=0.102\cdot \frac{2F}{\pi D^{2}\left(1-\sqrt{1-\frac{d^{2}}{D^{2}}}\right)}$$

with *d* being the mean diameter value of two indentations (*d*_1_, *d*_2_), measured at about 90° rotation.

## Ethics Statements

NA (i. e. work involves no human subjects, animal experiments or data collections from social media platforms)

## CRediT Author Statement

**Christian Rockenhäuser:** Investigation, Formal analysis, Validation, Visualization, Writing - Review & Editing; **Philipp von Hartrott:** Funding acquisition, Conceptualization, Supervision, Writing – Review & Editing; **Birgit Skrotzki:** Funding acquisition, Conceptualization, Resources, Supervision, Data Curation, Writing – Original Draft, Review & Editing.

## Declaration of Competing Interest

The authors declare that they have no known competing financial interests or personal relationships that could have appeared to influence the work reported in this paper.

## Data Availability

Brinell-Hardness (HBW 2.5/62.5) of Al-alloy EN AW-2618A after different aging times and temperatures (Original data) (Zenodo).
